# Disease patterns and clinical outcomes of patients admitted in intensive care units of tertiary referral hospitals of Tanzania

**DOI:** 10.1186/1472-698X-14-26

**Published:** 2014-09-23

**Authors:** Hendry R Sawe, Juma A Mfinanga, Salum J Lidenge, Boniventura CT Mpondo, Silas Msangi, Edwin Lugazia, Victor Mwafongo, Michael S Runyon, Teri A Reynolds

**Affiliations:** 1 Emergency Medicine Department, Muhimbili University of Health and Allied Sciences, Dar es salaam, Tanzania; 2 Department of Medical Oncology, Ocean Road Cancer Institute, Dar es salaam, Tanzania; 3 Department of Internal Medicine, University of Dodoma College of Health Sciences, Dodoma, Tanzania; 4 Department of Surgery, Kilimanjaro Christian Medical Centre, Moshi, Tanzania; 5 Department of Emergency Medicine and Global Health Sciences, University of California San Francisco, San Francisco, California, USA; 6 Department of Emergency Medicine, Carolinas Medical Center, Charlotte, NC, USA

## Abstract

**Background:**

In sub-Saharan Africa the availability of intensive care unit (ICU) services is limited by a variety of factors, including lack of financial resources, lack of available technology and well-trained staff. Tanzania has four main referral hospitals, located in zones so as to serve as tertiary level referral centers. All the referral hospitals have some ICU services, operating at varying levels of equipment and qualified staff. We analyzed and describe the disease patterns and clinical outcomes of patients admitted in ICUs of the tertiary referral hospitals of Tanzania.

**Methods:**

This was a retrospective analysis of ICU patient records, for three years (2009 to 2011) from all tertiary referral hospitals of Tanzania, namely Muhimbili National Hospital (MNH), Kilimanjaro Christian Medical Centre (KCMC), Mbeya Referral Hospital (MRH) and Bugando Medical Centre (BMC).

**Results:**

MNH is the largest of the four referral hospitals with 1300 beds, and MRH is the smallest with 480 beds. The ratio of hospital beds to ICU beds is 217:1 at MNH, 54:1 at BMC, 39:1 at KCMC, and 80:1 at MRH. KCMC had no infusion pumps. None of the ICUs had a point-of-care (POC) arterial blood gas (ABG) analyzer. None of the ICUs had an Intensive Care specialist or a nutritionist. A masters-trained critical care nurse was available only at MNH. From 2009–2011, the total number of patients admitted to the four ICUs was 5627, male to female ratio 1.4:1, median age of 34 years. Overall, Trauma (22.2%) was the main disease category followed by infectious disease (19.7%). Intracranial injury (12.5%) was the leading diagnosis in all age groups, while pneumonia (11.7%) was the leading diagnosis in pediatric patients (<18 years). Patients with tetanus (2.4%) had the longest median length ICU stay: 8 (5,13) days. The overall in-ICU mortality rate was 41.4%.

**Conclusions:**

The ICUs in tertiary referral hospitals of Tanzania are severely limited in infrastructure, personnel, and resources, making it difficult or impossible to provide optimum care to critically ill patients and likely contributing to the dauntingly high mortality rates.

## Background

The modern concept of intensive care is said to have been pioneered by an anesthetist in Denmark over half a century ago during the polio pandemic [1]. Since then, intensive care units (ICUs) have significantly improved the quality of care and outcomes of critically ill and injured patients, predominantly in high-resource settings [2-4].

In sub-Saharan Africa, ICUs have varying qualities and quantities of infrastructure necessary for the provision of proper critical cares services [5,6]. The reported disease characteristics and mortality rates of patients admitted to ICUs in sub-Saharan Africa vary widely from one population to another [7-9]. In a study of severe head injury patients in the ICU of National Hospital Abuja in Nigeria, the mortality rate was 68.4%, [10] while in another study of neurological and obstetric patients, the mortality rates were 43.5% and 33% respectively [11,12]. A similar study of critical care obstetric patients in Burkina Faso revealed a mortality rate of 60%, [13] while the mortality rate in the general ICU population in Uganda was found to be 25% [14].

**Figure 1:**
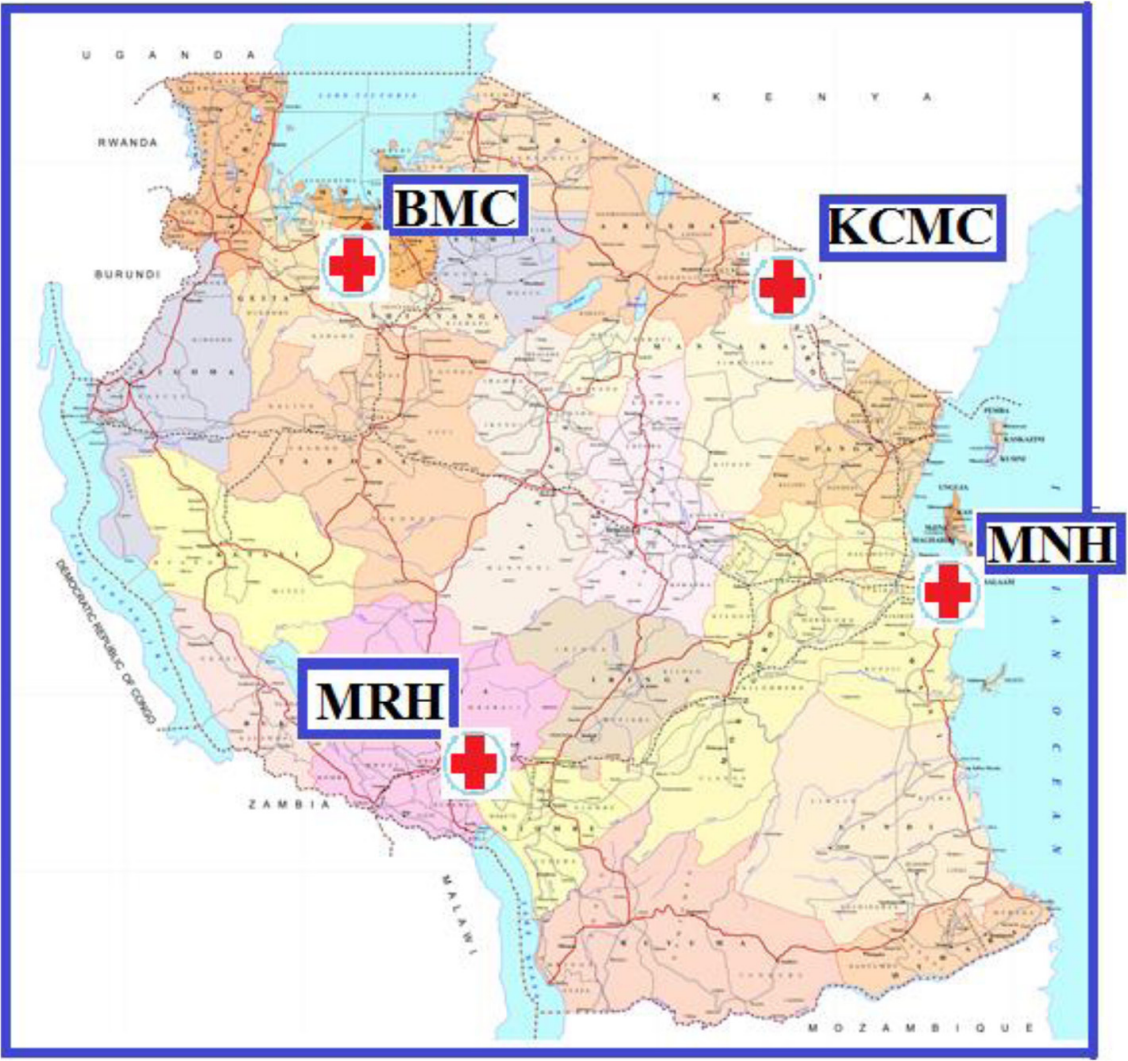
**Map of Tanzania showing location of the four referral hospitals.** Source: http://www.tanzania.go.tz; Accessed 26.06.2014.

Tanzania, a low-income country in East Africa with a population of 44 million, has four main referral hospitals, which are located in zones so as to serve as a tertiary level referral centers. These hospitals are Kilimanjaro Christian Medical Centre (KCMC) in the Northern zone, Bugando Medical Centre (BMC) in the Western zone, Mbeya Referral Hospital (MRH) in the Southern highlands zone, and Muhimbili National Hospital (MNH) which serves the coastal zone (Figure 1). MNH also serves as the National Referral Hospital, receiving patients from all hospitals in Tanzania, including the three named referral hospitals [15]. These are public access hospitals with government funding, and the only ones with specialized ICU care for patients with critical illness within the public system. In general, patients pay a standard fee scaled to income for hospital admission whether or not it also includes ICU admission; ability to pay is not a factor in deciding the ICU admission. A small number of private hospitals with semi-tertiary capacity provide specialized ICU care; however these services are not freely accessible to the general public.

All four referral hospitals have ICU services that operate with varying levels of equipment and staff. The regional hospitals in the respective zones usually send their sickest patients to these referral hospitals for ICU and other specialized care. The disease patterns and clinical outcomes of the patients admitted in the ICU of these four referral hospitals are unknown. In this study we aim to describe the disease patterns and clinical outcomes of patients admitted to the ICUs of these Tanzanian referral hospitals.

## Methods

This is a retrospective review of patients admitted to the ICUs of the four public tertiary referral hospitals of Tanzania (MNH, KCMC, BMC, and MRH) for the three year period from 1 January 2009 to 31 December 2011.

These ICUs are dedicated patient care areas at each of the four hospitals, specifically tasked with caring for critically ill and injured patients. They serve all such patients regardless of diagnosis (medical, surgical, trauma, etc.) and age (adult and paediatric).

Trained physician abstractors analyzed patient care log books, attendance registers, nurses' report books, and death certificates. Separate information was obtained on the basic characteristics of each of the ICUs, including human resources and available medical equipment. The information was recorded on a structured questionnaire that was developed by a consensus process among the investigators based on the elements from previous literature [6-8]. Study data, including basic demographics and primary diagnoses, were transferred from the hand-written data forms into an Excel spreadsheet (Microsoft Corporation, Redmond, WA, USA) and then manually coded into Clinical Classifications Software (CCS) multi-level categories (Agency for Healthcare Research and Quality, Rockville, MD, USA) [16]. All of the CCS categorizations were reviewed by at least two physician researchers, and any disagreements were mediated by a third physician.

Procedure, frequency, and univariate functions were performed to check for any outliers and to clean the dataset. The descriptive statistics including the total number of patients admitted to each ICU, gender distribution, and age groups were calculated. Additional study data included: 1) the frequencies and average length of ICU stay for each CCS diagnosis category; 2) the rates and causes of ICU deaths; and 3) the in-ICU mortality rates for each diagnoses, calculated as the number of deaths of patients given a particular diagnoses divided by the total number of patients with that diagnosis.

In addition we report the aggregate categories of Infectious disease, trauma, post-operative observation, disease of circulatory system and pregnancy related. Each aggregate category includes all of the relevant individual CCS multilevel diagnoses.

This retrospective analysis was approved by the Muhimbili University of Health and Allied Sciences (MUHAS) Institutional Review Board.

## Results

### Intensive care unit characteristics

MNH is the largest referral hospital with 1300 beds, while MRH is the smallest with 480 beds. MNH had the largest ratio of hospital to ICU beds (217:1) while KCMC had the smallest (39:1).

MNH and BMC had one cardiac monitor for each ICU bed, and KCMC had one monitor for three beds. There was one ventilator for four ICU beds at KCMC, while MNH had a one for each bed. MNH had one infusion pump for each bed, KCMC had none. None of the ICUs had a point-of-care (POC) arterial blood gas (ABG) analyzer or the ability to monitor central venous pressure (CVP).

BMC had the lowest physician-to-patients’ ratio (1:1146). The lowest nurse-to-patient ratio was observed at KCMC (1:77). None of the ICUs had an Intensive Care-trained medical specialist or a nutritionist. A critical care nurse was available only at MNH, and a respiratory therapist was available only at KCMC.

The availability of ICU resources is shown in Table 1.

**Table 1:** Intensive care unit characteristics

	**Hospitals**			
	**MNH**	**BMC**	**KCMC**	**MRH**
Hospital bed capacity	1300	650	540	480
**ICU RESOURCES**				
ICU bed capacity	6	12	14	6
Ventilators	6	5	4	4
Cardiac monitors	6	12	5	3
Oxygen supply ports	12	14	7	4
Invasive BP monitoring (arterial line)	0	0	0	0
CVP monitoring capacity	0	0	0	0
Arterial blood gas analyzer	0	0	0	0
Portable ultrasound	0	0	0	1
Portable X-ray	1	1	1	1
Infusion pumps	6	4	0	2
ECG machine	0	0	1	0
Renal replacement therapy	0*	0	0	0
Capnography monitoring capacity	0	0	0	0
**ICU STAFFING**				
Number of fellowship-trained intensivists	0	0	0	0
Number specialist anaesthesiologists	4	2	3	1
Number of masters-trained critical care nurses	1	0	0	0
Number of nutritionists	0	0	0	0
Number of respiratory therapists	0	0	1	0
Nurse to patient ratio**	1:2	1:4	1:4	1:2
Physician to patient ratio***	1:6	1:12	1:14	1:6

*Since the date of our study, MNH has introduced hemodialysis.

**The ratio is based on an 8 hour staffing coverage, all ICU follow an 8 hour shift system.

***The ratio is based on a daily coverage.

ICU-Intensive Care Unit.

CVP- central venous pressure.

ECG- Electrocardiography.

MNH-Muhimbili National Hospital.

BMC-Bugando Medical Centre.

KCMC-Kilimanjaro Christian Medical Centre.

MRH-Mbeya Referral Hospital.

### ICU patients’ demographics

During the study period, 5627 patients were admitted to ICU. Male patients comprised the majority of the study population, male: female ratio of 1.4:1. The median age of study population was 34 years, with an interquartile range of 21–53 years (Table 2).

**Table 2:** ICU patient demographics

		**Overall N = 5627**	**MNH N = 1154**	**BMC N = 2292**	**KCMC N = 1608**	**MRH N = 573**
Gender	Male	57.5%	55.5%	53.6%	57.9%	53.6%
	Female	41.8%	41.8%	45.8%	41.9%	45.8%
	No gender data	0.7%	2.7%	0.6%	0.2%	0.6%
Median age in years (Interquartile Range)		34 (21–53)	34 (24–52)	30 (15–48)	36 (21–52)	37 (24–56)
Age groups (Years)	<5	12.1%	6.2%	11.3%	6.4%	11.3%
	5-17	10.5%	15.0%	9.8%	9.5%	9.8%
	≥18-65	70.4%	72.1%	75.5%	73.3%	72.6%
	>65	5.6%	4.3%	3.2%	9.8%	6.1%
	No age data	1.4%	2.4%	0.2%	1.0%	0.2%
Overall In-ICU mortality			2331 (41.4%)			
N = 5627						
Overall length of ICU stay in days			5 [3,11]			
Median (Q1,Q3)						

MNH-Muhimbili National Hospital.

BMC-Bugando Medical Centre.

KCMC-Kilimanjaro Christian Medical Centre.

MRH-Mbeya Referral Hospital.

### Top ICU diagnoses and disease categories by age group

Intracranial injury was the most frequent admission diagnosis for all age groups. Post-operative observation, the second most frequent admission diagnosis had lowest in-ICU mortality.

In pediatric patients aged less than 5 years, non-TB pneumonia was the leading cause of ICU admission, and had the highest in-ICU mortality.

The top ICU diagnoses by age group are shown in Table 3.

**Table 3:** Top ICU diagnoses by age group

**Top 10 diagnoses for adults and all ages (including age unknown)**				
**CCS category**	**As % of all patients N = 5627**	**As % of adult patients (≥18 years) N = 4277**	**Length of ICU stay in days median (Q1,Q3) (All ages)**	**In-ICU mortality (All ages)**
Intracranial injury	703 (12.5%)	665 (15.5%)	5 (3,12)	280 (39.8%)
Post-operative observation*	597 (10.6%)	461 (10.8%)	1 (1,2)	73 (12.2%)
Injury and poisoning	400 (7.1%)	321 (7.5%)	4 (2,9)	189 (47.3%)
Other intestinal obstruction	360 (6.4%)	248 (5.8%)	3 (2,7)	141 (39.2%)
Acute but ill-defined cerebrovascular accident	360 (6.4%)	360 (8.4%)	6 (4,11)	247 (68.6%)
Complications of pregnancy; childbirth; and the puerperium	315 (5.6%)	253 (5.9%)	5 (2,9)	109 (34.6%)
Diabetes with ketoacidosis or uncontrolled diabetes	298 (5.3%)	259 (6.1%)	5 (2,7)	113 (37.9%)
Peritonitis and intestinal abscess	264 (4.7%)	221 (5.2%)	4 (2,9)	137 (51.9%)
Hypertension with complications and secondary hypertension	191 (3.4%)	191 (4.5%)	4 (2,7)	95 (49.7%)
Pneumonia (except that caused by TB)	174 (3.1%)	101 (2.4%)	3 (2,5)	130 (74.7%)
**Top 5 diagnosis in very young children (<5 years)**				
**CCS category**	**As % of age group N = 681**		**Length of ICU stay in days median (Q1,Q3)**	**In-ICU mortality**
Pneumonia (except that caused by TB)	86 (12.6%)		3 (1,10)	70 (81.4%)
Infectious and parasitic diseases**	69 (10.1%)		3 (1,9)	48 (69.6%)
Meningitis (except that caused by TB or STD)	65 (9.5%)		5 (3,11)	47 (72.3%)
Septicaemia (except in labor)	49 (7.2%)		4 (2,12)	39 (79.6%)
Post-operative observation*	41 (6.0%)		2 (1,4)	7 (17.1)

*Not a CCS category but was used for patients admitted to the ICU for Immediate post-surgical observation (after both elective and non-elective procedures).

**This category was used exclusively for Malaria cases, for which there is no disease-specific CCS category.

### Top disease categories by ICU for all ages

**Figure 2:**
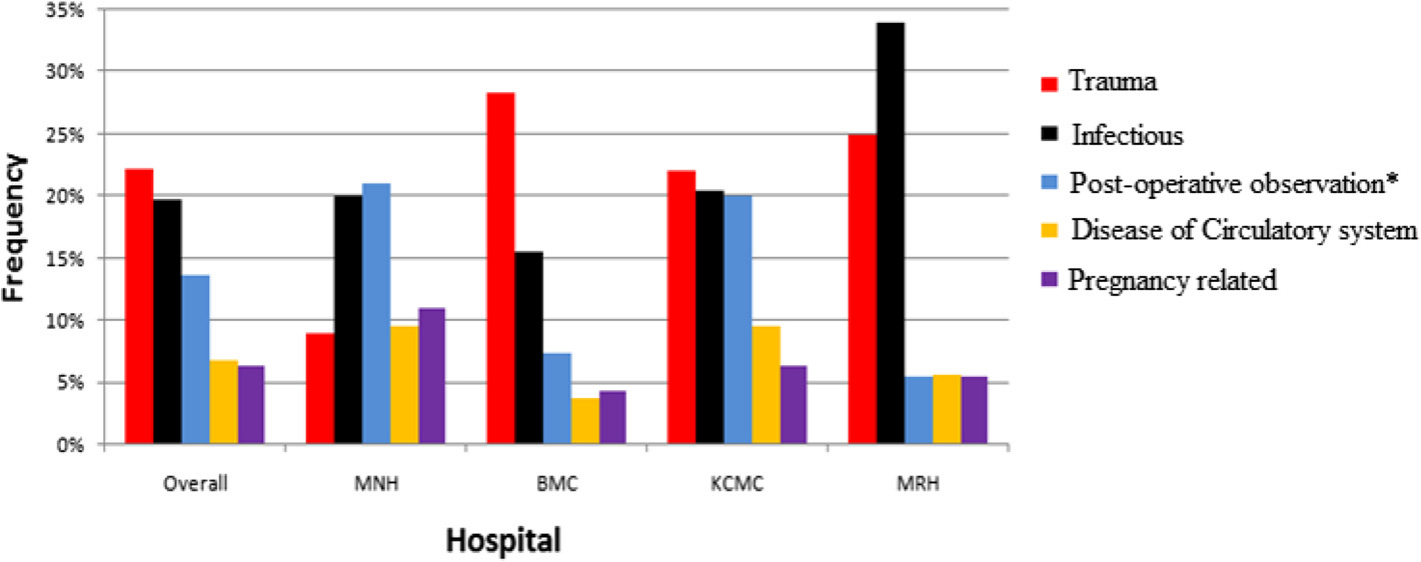
Top disease categories by ICU for all ages.

Non communicable disease categories (trauma, post-operative observation, disease of circulatory system and pregnancy related conditions) accounted for over half of the admission diagnoses (Figure 2).

### In-ICU mortality

The overall in-ICU mortality was 41.4% (Table 2). Among patients of all age groups, renal failure (acute and chronic), shock, burns and septicemia were conditions with the highest in-ICU mortality rate of over 80% each. Tetanus had the longest median of ICU stay, and an in-ICU mortality of 71.1%. In pediatric patients aged less than 5 years, non-TB pneumonia had the highest in-ICU mortality (Table 4).

**Table 4:** Conditions with highest in-ICU mortality

**Conditions with highest in-ICU mortality for all ages**			
**CCS category**	**As % of all patients N = 5627**	**Length of ICU stay in days median (Q1,Q3)**	**In-ICU mortality**
Chronic renal failure	23 (0.4%)	2 (1,4)	21 (91.3%)
Acute renal failure	51 (0.9%)	2 (1,3)	45 (88.2%)
Shock	90 (1.6%)	2 (1,5)	78 (86.7%)
Burns	56 (1.0%)	6 (2,9)	48 (85.7%)
Septicaemia (except in labor)	68 (1.2%)	4 (2,12)	55 (80.9%)
Pneumonia (except that caused by TB)	174 (3.1%)	3 (2,5)	130 (74.7%)
Other bacterial infections***	135 (2.4%)	8 (5,13)	96 (71.1%)
Acute but ill-defined cerebrovascular accident	360 (6.4%)	6 (4,11)	247 (68.6%)
Meningitis (except that caused by TB or STD)	140 (2.5%)	4 (3,13)	94 (67.1%)
Acute myocardial infarction	45 (0.8%)	5 (4,9)	27 (60.0%)
**Top 5 pediatric (<5 years) conditions with highest in-ICU mortality**			
**CCS category**	**As % of age group N = 681**	**Length of ICU stay in days median (Q1,Q3)**	**In-ICU mortality**
Pneumonia (except that caused by TB)	86 (12.6%)	3 (1,10)	70 (81.4%)
Septicaemia (except in labor)	49 (7.2%)	4 (2,12)	39 (79.6%)
Meningitis (except that caused by TB or STD)	65 (9.5%)	5 (3,11)	47 (72.3%)
Infectious and parasitic diseases**	69 (10.1%)	3 (1,9)	48 (69.6%)
Fluid and electrolyte disorders	15 (2.2%)	3 (2,6)	10 (66.7%)

**All were Malaria cases, for which there is no specific CCS category.

***All were Tetanus cases, for which there is no specific CCS category.

## Discussion

In this retrospective review, our findings of very high relative mortality and the substantial essential resource gaps suggests that all four tertiary referral hospitals of Tanzania significantly lack the necessary resources and infrastructure for the provision of high-quality intensive care to critically ill patients. Similar to the published literature on the state of ICUs in other developing countries [7,8,16-19] we found that none of the ICUs components reviewed met the minimum standards of basic requirements of an intensive care unit, as per multi-national consensus or professional bodies recommendations [2,20].

In all ICUs, the percentage of beds is far below internationally recommended standard of 5-10% of the overall hospital beds [21-24]. Half of the ICUs had less than one cardiac monitor per bed, none had CVP monitoring capacity for volume assessment in patients, and all ICUs lack the ability to perform bedside ABG analysis, a core component of care in critically ill and ventilated patients [25,26]. This shortage poses an enormous challenge to the care of critically ill patients, and though this was not the subject of our study, per discussions with providers in each of these hospitals, there is a general conviction that ICU care is not available for many patients who need it.

There is a significant shortage of intensive care providers in all the ICUs; none of the ICUs have fellowship-trained critical care specialists, and only one hospital has a master’s-trained critical care nurse. The care of patients in the ICU is therefore dependent on just a few available anesthesiologists, internists, or surgeons, while nursing care is provided by ordinary nurses without formal critical care training. These findings are similar to a recent survey of a convenience sample of 10 regional hospitals in Tanzania, which found a significant lack of critical care infrastructure, resources and training [27].

The patient population in this study is younger compared with patients admitted to ICUs in most of developed world [28,29], a similar demographic finding to other studies of ICUs in sub-Saharan Africa which have reported a predominantly young-age ICU population [8,22,30,31]. The overall young population and male predominance in our study, may in some extent, reflect the prevalence of trauma, long shown to have a working age male predominance [32]. Another factor that may also affect median age is the admitting providers’ views on the prognosis of older patients; however, we did not address admission decision making in our study.

Paediatric patients under the age of five years accounted for 12.1% of the study population, a modest volume, but we suspect this proportion is still artificially low based on prior studies of critically ill-children in our settings [31,33,34]. This statistic reflects a severe shortage of dedicated, age-appropriate ICU capacity, even at the top level referral hospitals in Tanzania.

Non-communicable disease conditions accounted for most of the ICU admissions, with trauma alone constituting over twenty percent, highlighting the growing burden of non-communicable diseases within Sub-Saharan Africa, as shown in previous studies [35,36]. The regional practice of restricting admission of potentially infectious patients to ICU units may have also resulted in the triage of even critically ill patients with communicable disease to lower levels of care, inflating the proportion of non-communicable diseases in the ICU, For example, all of the ICU in Tanzania place some limits on admission of patients with TB, HIV or Hepatitis C, regardless of acuity.

Intracranial injury was the most common reason for ICU admission in both adult and overall populations and carried a substantial in-ICU mortality, similar to findings in previous studies in other developing countries [30,37,38]. Among the top ten ICU diagnoses, patients with non-TB pneumonia had the highest in-ICU mortality, potentially because these were HIV/AIDS patients with pneumocystis jiroveci pneumonia (PCP), known to carry a high in-ICU mortality, even in area of high resources [39].

In pediatric patients aged less than 5 years, non-TB pneumonia was the commonest reason of ICU admission and carried the highest in-ICU mortality. This observation is consistent with previous studies indicating both the high prevalence and acuity of pediatric pneumonia patients from similar settings [40,41].

The overall in-ICU mortality rate of 41.4%, is comparable to other studies in Africa [30], but much higher than mortality reports in developed countries [28,42]. Similarly, the median length of ICU stay in our study is also comparable to that of ICUs in other parts of Sub-Saharan Africa [30]. Among patients of all age groups, chronic renal failure, acute renal failure, shock, burns, and septicemia each had in-ICU mortality rates of over eighty percent. During the study period, none of the referral hospitals were offering dialysis services for renal failure patients, a fact that may partially account for such high mortality rate observed. Lack of dedicated burn ICU personnel and resources, along with likelihood of high severity (high percentage burns, deep burns, inhalational burns, or sepsis) of patients admitted in ICUs may explain the reason for high in-ICU mortality among burn patients. Finally, like previous studies done in similar settings [43], patients with tetanus had the longest median length of ICU stay, and carried an in-ICU mortality of around 71%.

### Limitations

The limitations of this study include the retrospective design and the relative paucity of clinical data available in the attendance registers, nurses' report books, and death certificates.

The available data only supported categorization of patients by the primary diagnosis and the specific means by which the clinicians arrived at theses diagnoses is unclear. Also, the lack of referral data on patients admitted to the ICU limits our ability to speculate on the mortality impact due to lack of access to critical care in district and regional hospitals referring patients to these centers. Physiologic data necessary to calculate severity of illness or injury, such as the Apache score or Injury Severity Score, were lacking. Likewise, this study provides quantitative data on the available ICU personnel and resources, but not on the specific quality of care delivered. Owing to these limitations we did not feel it appropriate to make direct comparisons of mortality across the four study centres.

## Conclusion

This study clearly demonstrates that the ICUs in tertiary referral hospitals of Tanzania are severely limited in infrastructure, personnel, and resources, making it difficult or impossible to provide optimum care to critically ill patients and likely contributing to the dauntingly high mortality rates. While further research is necessary to characterize standardized severity scores of ICU patients and measure the quality of care delivered in these units to allow for bench-marking and comparison between facilities, health policy initiatives should focus on increasing ICU capacity in Tanzania.

## Abbreviations

ICU: Intensive care unit; CVP: Central venous pressure; ECG: Electrocardiography; MNH: Muhimbili National Hospital; BMC: Bugando medical centre; KCMC: Kilimanjaro Christian medical centre; MRH: Mbeya referral hospital.

## Competing interests

The authors declare that they have no competing interests.

## Authors’ contributions

HS contributed to the conception and design of the study, acquired, analyzed and interpreted the data, and drafted and revised the manuscript. JM contributed to the design of the study, data acquisition and entry and also revised the manuscript. SJ contributed to the design of the study, data acquisition and revised the manuscript. BM contributed to the design of the study and critically revised the manuscript. SM contributed to the design of the study, data acquisition and revised the manuscript. VM contributed to the conception and assisted in the initial design of the study and critically revised the manuscript. EL contributed to the conception and assisted in the initial design of the study and critically revised the manuscript. TR contributed to the conception and assisted in the initial design of the study, data interpretation and critically revised the manuscript. MR contributed to the conception and assisted in the initial design of the study, analyzed and interpreted the data and critically revised the manuscript. All authors read and approved the final manuscript.

## Pre-publication history

The pre-publication history for this paper can be accessed here:

http://www.biomedcentral.com/1472-698X/14/26/prepub
